# Long-Term Engraftment of Human Natural T Regulatory Cells in NOD/SCID IL2rγc^null^ Mice by Expression of Human IL-2

**DOI:** 10.1371/journal.pone.0051832

**Published:** 2012-12-18

**Authors:** Sojan Abraham, Rajendra Pahwa, Chunting Ye, Jang-gi Choi, Savita Pahwa, Shashidhar Jaggaiahgari, Ashwin Raut, Shuiping Chen, N. Manjunath, Premlata Shankar

**Affiliations:** 1 Department of Biomedical Sciences, Center of Excellence in Infectious Disease Research, Paul L. Foster School of Medicine, Texas Tech University Health Sciences Center, El Paso, Texas, United States of America; 2 Max Healthcare Super Specialty Hospital and Max Research Institute 1, Saket New Delhi, India; 3 Developmental Center for AIDS Research, Microbiology and Immunology, University of Miami, Leonard M. Miller School of Medicine, Miami, Florida, United States of America; University of Southern California, United States of America

## Abstract

Regulatory T cells are essential to maintain immune homeostasis and prevent autoimmunity. Therapy with *in vitro* expanded human nT_Regs_ is being tested to prevent graft versus host disease, which is a major cause for morbidity and mortality associated with hematopoietic stem cell transplantation. Their usefulness in therapy will depend on their capacity to survive, migrate appropriately and retain suppressive activity when introduced into a transplant recipient. The lack of a suitable animal model for studying the *in vivo* reconstitutive capability of human nT_Regs_ is a major impediment for investigating the behavior of adoptively transferred nT_Regs_ *in vivo*. We show that injection of a plasmid encoding human IL-2 is necessary and sufficient for long term engraftment of *in vitro* expanded nT_Regs_ in NOD-SCID IL2rγc^null^ mice. We also demonstrate that these *in vivo* reconstituted T_Regs_ traffic to different organs of the body and retain suppressive function. Finally, in an IL-2 accelerated GVHD model, we show that these *in vivo* reconstituted T_Regs_ are capable of preventing severe xenogenic response of human PBMCs. Thus, this novel ‘hu-T_Reg_ mouse’ model offers a pre-clinical platform to study the *in vivo* function and stability of human nT_Regs_ and their ability to modulate autoimmune diseases and GVHD.

## Introduction

Naturally arising T regulatory cells (nT_Regs_) which originate in the thymus are a subset of CD4^+^ T cells, which are critical both for suppressing autoreactive lymphocyte responses and for preventing exaggerated antigen-specific immune responses. Their importance is clearly illustrated by lethal systemic autoimmunity and lymphoproliferative disease observed in humans with mutated forkhead box P3 transcription factor (Foxp3) gene and in Foxp3-deficient mice [1], [2], [3]. nT_Regs_ are characterized by the co-expression of Foxp3 and interleukin-2Rα chain CD25. Another distinguishing feature is their dependence on exogenous interleukin-2 (IL-2) for growth and function [4]. With increased understanding of T_Reg_ biology and function, there has been a surge of interest in developing T_Reg_-based cellular therapy for a variety of immunological diseases in humans, most notably to prevent graft rejection and reduce the severity of graft versus host disease (GVHD), a frequent and often severe complication following allogenic hematopoietic cell transplantation. The major limitation for T_Reg_-based immunotherapy is their low number in peripheral blood, which makes it necessary to develop a robust method for large-scale expansion *in vitro*. The other major challenge is to ensure that *in vitro* expanded T_Regs_ are not contaminated with conventional T cells that could potentially exacerbate inflammatory response in the transplantation setting. We and others have shown earlier that purified nT_Regs_ can be expanded *in vitro* to clinically relevant numbers without loss of the signature CD25^+^Foxp3^+^ expression. Using anti-CD3/CD28 expander dynabeads and IL-2 in presence of rapamycin, we were able to achieve hundred-fold expansion of nT_Regs_ that retained their phenotype and suppressive function with no evidence of conversion to inflammatory effector or Th17 T cells [5].

Successful use of human T_Regs_ to suppress GVHD and graft rejection has recently been reported in humanized mouse models. Infusion of *in vitro* expanded human T_Regs_ together with PBMCs could significantly reduce GVHD in NOD/SCID and NOD-SCID IL2rγc^null^ mice [6], [7]. Further, it was shown recently that expanded T_Regs_ are effective in abrogating the development of transplant arteriosclerosis (TA) in a humanized mouse model [8]. Another study demonstrated the utility of cultured T_Regs_ in preventing allograft rejection in a human skin graft model in BALB/c Rag IL2rγc^null^ mice [9]. Despite these encouraging results, translation to efficacy in humans still remains uncertain. Success in using *in vitro* expanded T_Regs_ for immunosuppressive therapy in humans will depend on their capacity to survive, retain their phenotype, migrate appropriately and exert stable suppressive activity when introduced into the transplant recipient. Currently, there is no suitable model system to investigate the fate and function of human nT_Regs_ *in vivo*. A preclinical model in which expanded human nT_Reg_ reconstitution, trafficking, stability and function can be studied systematically will allow in depth studies of their *in vivo* behavior and set the stage for testing novel approaches to manipulate the cells for more optimal therapeutic results. In this report, we show that *in vitro* expanded human nT_Regs_ can be reconstituted in NOD-SCID IL2rγc^null^ mice by inducing the expression of IL-2 *in vivo* via hydrodynamic injection of hIL-2 expressing plasmid. Moreover, the reconstituted T_Regs_ retained their characteristic phenotype as well as suppressive function and were able to traffic to various organs including liver, spleen and lungs. Finally, these *in vivo* reconstituted T_Regs_ were capable of preventing severe xenogenic response of human PBMCs in an IL-2 accelerated GVHD model.

## Results

### Human IL-2 Expression by Hydrodynamic Injection of IL-2 Encoding Plasmid DNA Allows *in vivo* Expansion of Infused T_Regs_ in NOD-SCID IL2rγc^null^ Mice

**Figure 1:**
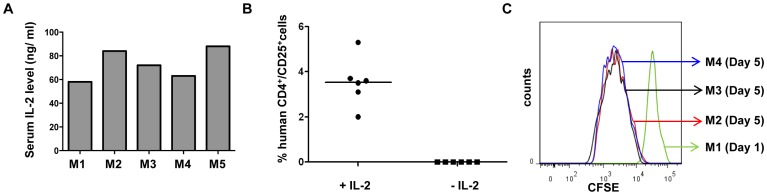
hIL-2 is required for *in vivo* reconstitution of nT_Regs_ in NOD-scid IL2rγc^null^ mice. (A) NOD-scid IL2rγc^null^ mice were hydrodymanically injected with human IL2 encoding plasmid and after 24h, their sera were tested for hIL2 by ELISA. (B) hIL-2 expressing or control NOD-scid IL2rγc^null^ mice were iv injected with 5×10^6^ *in vitro* expanded nT_Regs_. On day 12 MNCs from spleen were analyzed for presence of human CD4^+^ CD25^+^ cells on human CD45 gated population. Each symbol represents an individual mouse and horizontal bars indicate the mean values. (C) 5×10^6^ CFSE labeled T_Regs_ were iv injected into hIL-2 expressing NOD-scid IL2rγc^null^ mice and spleen cells obtained 1 (M1) or 5 days (M2–M4) later were analyzed for CFSE dilution on human CD4 gated population.

IL-2 signaling is required for both thymic development and peripheral expansion/maintenance of T_Regs_ [4]. T_Regs_ themselves do not produce this cytokine, so they have an obligatory requirement for IL-2 produced by other cells [4]. Large-scale *in vitro* expansion of polyclonal nT_Regs_ after stimulation with CD3/CD28 antibodies is also dependent on provision of exogenous IL-2. Thus, although the lymphopenic environment in NOD-SCID IL2rγc^null^ mice is considered conducive to homeostatic expansion of transferred human T cells, unlike conventional T cells, T_Regs_ are unlikely to engraft in the absence of an exogenous source of IL-2. The cytokine could be provided by administration of recombinant IL-2. However, the *in vivo* half life of the cytokine is in the range of minutes which would necessitate repeated injections for sustained action [10]. On the other hand, hydrodynamic delivery of plasmids expressing cytokine genes has been recently shown to be a simple, efficient and inexpensive method to express human cytokines in mice [11]. We injected hIL-2 expressing plasmid into these mice by hydrodynamic injection one day prior to nT_Regs_ infusion. *In vivo* expression of IL-2 was confirmed by measuring cytokine levels in the serum by ELISA after 24 hr **(** Figure 1A **)**.

As shown in our previous study, nT_Regs_ expanded in culture with CD3/CD28 stimulation in presence of IL-2 and rapamycin maintained their phenotypic and functional integrity (Figure S1A, B). To test reconstitution of the cells *in vivo*, we injected 5×10^6^ expanded nT_Regs_ into NOD-SCID IL2rγc^null^ mice in the presence or absence of hIL-2. The animals were sacrificed on day 12 and analyzed for reconstituted human cells in the spleen. MNCs isolated from spleen were stained with anti-human CD45, CD4 and CD25 antibodies. Our results show that human cells identified by expression of the three markers were present only in animals expressing human IL-2 (Figure 1B). The results demonstrate that provision of the cytokine from an exogenous source is essential for T_Reg_ reconstitution in NOD-SCID IL2rγc^null^ mice.

Next, we asked whether repopulation reflected just IL-2-dependent survival of the infused T_Regs_ or their further expansion *in vivo*. For this, we labeled *in vitro* expanded nT_Regs_ with CFSE dye just before injecting them into IL-2 expressing animals. The animals were sacrificed at day 1 or day 5 and cells isolated from spleen were assessed for CFSE dilution by flow cytometry. As shown in the Figure 1C, there was a substantial dilution of the dye on day 5 as compared to day 1, suggesting that the parent cells underwent cell division *in vivo*.

### T_Regs_ Maintain Stable Phenotype and Trafficking Capabilities after *in vivo* Reconstitution

**Figure 2:**
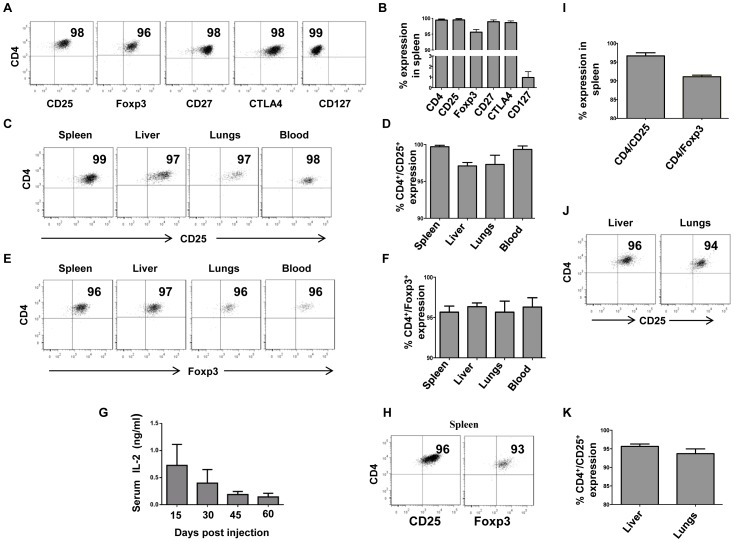
Reconstituted T_Regs_ retain their phenotypic characteristics and traffic to different organs of NOD-scid IL2rγc^null^ mice. (A, B) 5×10^6^ expanded nT_Regs_ were iv injected into NOD-scid IL2rγc^null^ mice in the presence hIL-2. On Day 12, MNCs were isolated from the spleen and surface stained for CD4, CD25, CD27, CD45 and CD127 and then intracellularly stained for CTLA4 and Foxp3. Representative flow cytometric analysis of human CD45 gated population from one mouse (A) and composite data (B) from all the three animals tested are shown. (C, D, E, F) MNCs from indicated organs were tested for T_Reg_ reconstitution by examining for CD4 and CD25 (C, D) and Foxp3 (E, F) expression. Representative results from one mouse (C, E) and composite data (D, F) from all the three animals analyzed are shown. (G) Serum IL-2 levels at different time points after a single hydrodynamic injection of the plasmid were tested by ELISA. (H, I, J, K) 33 days after T_Reg_ reconstitution, MNCs purified from spleen, liver and lungs were analyzed for CD4, CD25 and Foxp3 expression on human CD45^+^ gated cells. Representative results from one mouse (H, J) and composite data (I, K) from all animals tested are shown. The numbers in the panel depict percentage of positive cells within human CD45 gated populations and error bars indicate standard deviation of three different animals tested.

To determine whether expanded nT_Regs_ maintain their phenotypic characteristics after *in vivo* reconstitution, we assessed the expression of multiple T_Reg_-associated markers, including CD4, CD25, Foxp3, CD127, CTLA4 and CD27 on human cells isolated from the spleens12 days after reconstitution. We found the expression pattern of these markers to be identical to the parent nT_Reg_ culture that was infused into the animals (Figure 2A, B and Figure S1A respectively).

The usefulness of T_Reg_ therapy would also rely on their ability to efficiently traffic to different organs where their suppressive activity may be required. Thus in addition to spleen, we also evaluated liver, lungs and blood for the presence of transferred human T_Regs_ 12 days after reconstitution. T_Regs_ reconstitution was analyzed as before, by staining the cells with anti-human CD45, CD4 and CD25 antibodies. Our results show that, the reconstituted T_Regs_ were present in spleen, liver, lungs and blood (Figure 2C, D). We also confirmed that these cells continue to express Foxp3 in different organs (Figure 2E, F). These data demonstrate that the reconstituted T_Regs_ have the capability to traffic to different organs of the body to exert their regulatory activity.

To determine how long IL-2 from a single hydrodynamic plasmid injection could sustain T_Reg_ reconstitution, we first measured IL-2 levels at various time points after plasmid injection. As shown in the Figure 2G, IL-2 could be detected in the serum for up to 60 days, although the levels declined somewhat at later time points. Correspondingly, human T_Reg_ reconstitution was sustained in the spleen, liver and lungs for up to 33 days when the experiment was terminated and these cells continued to express Foxp3 (Figure 2H–K).

### *In vivo* Reconstituted T_Regs_ do not Convert to Th17 Cells and Retain their Characteristic Suppressive Function

**Figure 3:**
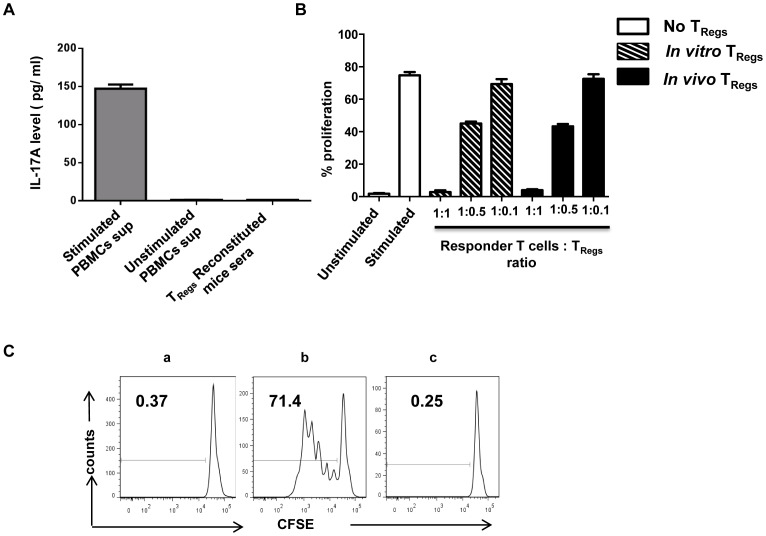
*In vivo* expanded T_Regs_ do not convert to Th17 cells and retain their characteristic suppressive function. (A) IL-17A levels in the sera of nT_Reg_ reconstituted animals were analyzed by ELISA. Error bars indicate standard deviation for six different animals tested. Supernatants from normal human PBMCs stimulated with PMA (50 ng/ml) and ionomycin (1 µg/ml) served as positive control. (B) T_Regs_ obtained from reconstituted mice and *in vitro* expanded T_Regs_ were tested for their ability to suppress proliferation of autologous CD4^+^CD25^−^ T cells at different T_Reg_ to CD4^+^CD25^−^ responder T cell ratios. Responder cells were stimulated with anti-CD3/CD28 beads and CFSE dilution was analyzed after 5 days of culture under the following conditions: autologous T cells cultured in medium without stimulation (unstimulated), stimulated with anti-CD3/anti-CD28 coated micro beads in the absence of T_Regs_ (stimulated), in the presence of cultured T_Regs_ (*in vitro* T_Regs_) or in the presence of T_Regs_ isolated from reconstituted mice 12 days after reconstitution (*In vivo* T_Regs_). Composite data from all the three animals tested are shown. (C) The functional stability of T_Regs_ obtained 33 days after *in vivo* reconstitution was also analyzed by CFSE dilution of CD4^+^CD25^−^ responder cells. Representative data from one of two animals tested is shown. Data depicts CD4^+^CD25^−^ responder cells cultured with medium alone (a), stimulated in the absence of *in vivo* T_Regs_ (b) and stimulated in the presence of *in vivo* T_Regs_ (c).

*In vitro* studies have shown that human T_Regs_ can be reprogrammed to IL-17A secreting Th17 cells in the presence of IL-2 and Th17 polarizing conditions [12]. It was also shown that culture of ovarian cancer associated T_Regs_ in presence of IL-2 resulted in their conversion to Th17 cells [13]. Based on these results, we also evaluated the possibility of *in vitro* expanded T_Regs_ converting to Th17 cells after *in vivo* reconstitution in the presence of IL-2. As shown in the Figure 3A, we did not find detectable levels of IL-17A in the serum obtained 12 days after nT_Reg_ reconstitution, which rules out their possible conversion to inflammatory Th17 cells under the influence of IL-2 *in vivo*. Moreover, there was no detectable level of IL-17A even in serum obtained 33 days post nT_Reg_ reconstitution (data not shown).

The ability of transfused T_Regs_ to retain their suppressive function *in vivo* would be the ultimate determinant for their usefulness as cellular therapy. To evaluate suppressive function of *in vivo* reconstituted T_Regs_, human T cells were purified from spleen harvested from animals on day 12 after reconstitution of human nT_Regs_. The isolated cells were tested for their ability to inhibit the proliferative response of autologous CD4^+^CD25^−^ responder cells to CD3/CD28 stimulation, as this is used as the classical assay to assess suppression mediated by T_Regs_ [14]. Autologous CD4^+^CD25^−^ responder T cells were labeled with CFSE dye and mixed with T_Regs_ isolated from *in vivo* reconstituted mice or *in vitro* cultured T_Regs_ at a 1∶1 ratio, stimulated with CD3/CD28 and cultured for 5 days. Proliferation as determined by CFSE dilution with cell division was analyzed on day 5. As shown in Figure 3B, *in vivo* expanded T_Regs_ retained potent suppressive activity as they were able to inhibit the proliferation of the responder CD4^+^CD25^−^ T cells as efficiently as the parental nT_Regs_ cells used for infusion. Furthermore, the suppressive capability of T_Regs_ remained intact up to 33 days post transfer (Figure 3C).

### *In vivo* Expression of IL-2 Accelerates Xenogenic GVHD

**Figure 4:**
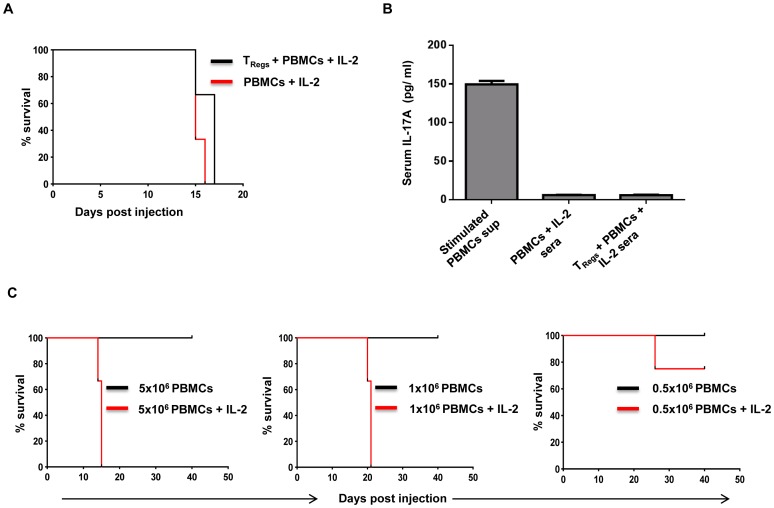
*In vivo* expression of hIL-2 accelerates the induction of xenogenic GVHD in the presence or absence of T_reg_ reconstitution. (A) Kaplan-Meir survival curve comparing IL-2 conditioned NOD-scid IL2rγc^null^ mice that received 5×10^6^ hPBMCs with or without prior reconstitution with 5×10^6^ T_Regs_ 12 days before injection with PBMCs. (B) IL-17A levels in the sera collected from the animals at the time of euthanasia. Error bars indicate standard deviation for three different animals tested. Supernatants from normal human PBMCs stimulated with PMA (50 ng/ml) and ionomycin (1 µg/ml) was used as positive control. (C) Kaplan-Meir analysis for survival of NOD-scid IL2rγc^null^ mice injected with graded doses of human PBMCs in presence of IL-2. Control and IL-2 conditioned animals were injected with different doses of hPBMCs (5×10^6^, 1×10^6^ and 0.5×10^6^) and observed for GVHD symptoms. Each group consisted of 3 mice.

IL-2 therapy *in vivo* has been suggested to favor immunosuppression by T_Regs_ rather than immune enhancement of effector T cells. Based on this premise, we evaluated the ability of NOD-SCID IL2rγc^null^ mice reconstituted with nT_Regs_ in the presence of IL-2 to resist GVHD induced by injected human PBMCs. After hydrodynamic injection with plasmid expressing hIL-2, animals were either reconstituted with 5×10^6^ nT_Regs_ as described earlier or not reconstituted to be used as controls. After 10 days, both groups were injected with 5×10^6^ allogenic human PBMCs by the iv route and observed for symptoms of GVHD over time. Contrary to expectations, animals with reconstituted T_Regs_ were not protected. In fact, compared to what has been reported for xenogenic GVHD by human cells in the absence of exogenous IL-2, here we observed an accelerated course of GVHD in both groups. Previous studies have reported that NOD-SCID IL2rγc^null^ mice reconstituted with human PBMCs without IL-2 remain free of GVHD symptoms for nearly 30 days after engraftment even with the transfer of up to 20×10^6^ PBMCs [15] whereas, here with just 5×10^6^ cells, all of the animals succumbed to the disease by 15–17 days ( Figure 4A). No IL-17A was detected in the sera of animals injected with PBMCs with or without prior T_Regs_ engraftment (Figure 4B), ruling out the possibility that the lack of protection was because of conversion of the *in vivo* expanded n T_Regs_ to Th17 cells in the GVHD setting.

We then determined the effect of human IL-2 in the initiation and progression of GVHD in NOD-SCID IL2rγc^null^ mice at different doses of PBMCs. Animals were injected with 5×10^6^, 1×10^6^ and 0.5×10^6^ PBMCs in the presence or absence of human IL-2. As shown in Figure 4C, the presence of IL-2 dramatically altered the course of the disease, with all the animals developing GVHD with 5×10^6^ PBMCs and 1×10^6^ PBMCs by 15–16 and 21–23 days respectively. Even with 0.5×10^6^ PBMCs 25% of the animals developed GVHD by day 30. None of the animals reconstituted in the absence of IL-2 developed signs and symptoms of GVHD until the experiment was terminated at day 40.

### Co-injection of *in vivo* Expanded T_Regs_ with Human PBMCs at 1∶1 Ratio Protects NOD-SCID IL2rγc^null^ Mice from IL-2 Accelerated GVHD

**Figure 5:**
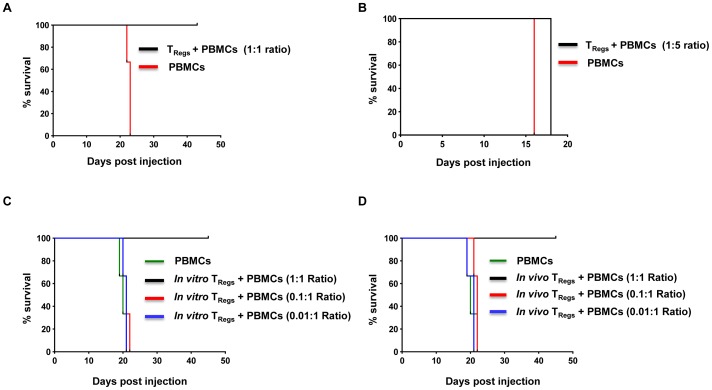
*In vivo* expanded T_Regs_ co-transplanted with hPBMCs are capable of preventing IL-2 accelerated GVHD in NOD-scid IL2rγc^null^ mice. (A, B) Kaplan-Meir curve showing the survival of IL-2 conditioned NOD-scid IL2rγc^null^ mice injected with hPBMCs alone or hPBMCs along with *in vivo* expanded T_Regs_. T_Regs_ purified from animals 12 days after reconstitution were used for co-transfer along with hPBMCs at 1∶1 (A) or 1∶5 (B) ratio of T_Regs_:PBMCs and the animals were observed for GVHD symptoms. (C, D) Kaplan-Meir curve comparing protection from GVHD conferred by *in vitro* and *in vivo* expanded T_Regs_. IL-2 conditioned NOD-scid IL2rγc^null^ mice were injected with hPBMCs only or co-transferred with different ratios of *in vitro* (C) or *in vivo* (D) expanded T_Regs_ and hPBMCs. In all experiments, each group consisted of 3 mice.

The inability of circulating T_Regs_ in reconstituted animals to prevent IL-2 accelerated GVHD symptoms could be due to insufficient T_Reg_ numbers as T_Regs_ are known to reliably silence GVHD in murine models only when used at the optimal dose [16]. To test the possibility that the lack of protection we observed in the IL-2 accelerated GVHD model was due to insufficient number of T_Regs_ and not due to dysfunction of the *in vivo* expanded cells, we first tested protection from GVHD induced by transfer of 1×10^6^ PBMCs in animals reconstituted with 5×10^6^ T_Regs_ 10 days earlier. However, here again, the control as well as the test group of animals succumbed to the disease (data not shown). To understand whether this implied T_Regs_ dysfunction *in vivo*, we tested the efficacy of reconstituted T_Regs_ in a co-transfer experiment, in which 1×10^6^ PBMCs obtained from a normal donor were adoptively transferred iv into new IL-2 conditioned animals along with T_Regs_ isolated from the spleens of mice 12 days after reconstitution at a 1∶1 or 1∶5 (T_Regs_: PBMCs) ratio. As only 0.2–0.4 million T_Regs_ could be recovered from the spleen of each reconstituted animal, the cells were pooled from multiple animals to achieve the requisite numbers for co-transfer. As shown in Figure 5A and 5B, all the control animals receiving PBMCs only or those co-injected with T_Regs_ at a 1∶5 (T_Reg_: PBMCs) ratio developed severe symptoms and had to be euthanized by around 16–21 days, whereas animals co-injected with T_Regs_ at a 1∶1 ratio were protected until day 45, when the experiment was terminated. The spleen and liver of diseased animals showed gross enlargement and flow cytometric analysis of cells isolated from the organs showed large-scale expansion of human CD4^+^ and CD8^+^ T cells. On the other hand in protected animals, the organs appeared to be of normal size and showed very low numbers of human cells (data not shown).

We also directly compared the *in vivo* potency of reconstituted T_Regs_ with the cultured parent n T_Regs_ in a separate experiment. Human PBMCs were transferred into IL-2 conditioned animals in the presence of various ratios of *in vitro* or *in vivo* expanded T_Reg_ (1∶0.01, 1∶0.1 and 1∶1 PBMC: T_Reg_ ratio). As shown in Figures 5C and 5D, in both groups, animals that received lower numbers of T_Regs_ succumbed to GVHD, whereas animals co-injected with T_Regs_ at 1∶1 ratio were protected until day 45, when the experiment was terminated. The equivalent protection observed with both populations also makes it unlikely that there was any significant conversion of T_Regs_ to proinflammatory cells in presence of IL-2 *in vivo* either at the first reconstitution or after adoptive transfer into fresh animals. Further, the lack of protection at the lower T_Reg_:PBMC ratio suggests that optimal numbers of T_Regs_ are required for effective protection. As the number of *in vivo* expanded T_Regs_ required for protection could only be achieved by pooling cells from multiple animals, the limited expansion of the cells *in vivo* may be a major factor for their inability to protect in the non co-transfer setting. The other possibility is that to exert their suppressive function, T_Regs_ require cell: cell contact which is likely to be limiting when PBMCs are injected separately into T_Regs_ reconstituted animals.

Protection from IL-2 accelerated GVHD at the higher 1∶1 (T_Reg_: PBMC) ratio could also imply that the T_Regs_ are preferentially using up all of the IL-2 produced *in vivo*. To rule out this possibility, in a separate experiment we also determined whether the same number of T_Regs_ (1×10^6^) could prevent GVHD induction when co-transferred at a 1∶5 ratio with 5×10^6^ PBMCs. In this setting, all of the animals exhibited GVHD symptoms by around 16–18 days (data not shown), suggesting that there is enough of the cytokine to support the massive expansion of large numbers (5×10^6^) of xenogenic PBMCs despite the presence of T_Regs_ injected alongside.

## Discussion

Induction of tolerance to allogeneic donor grafts is a clinically desirable goal in bone marrow and solid organ transplantation. As T_Regs_ play a major role in tolerance induction, infusion of *in vitro* expanded T_Regs_ is being developed as a novel therapeutic strategy to reduce the severity of GVHD and autoimmune diseases [17], [18]. Generating sufficient numbers of T_Regs_ is no longer a constraint for their therapeutic use as methods have been developed for *in vitro* expansion of enriched T_Regs_ without the loss of suppressive function [5], [7]. However, the lack of a preclinical animal model has been an impediment for gaining an improved understanding of their stability, trafficking capability and ability to expand *in vivo* without conversion to proinflammatory cells which are important issues for T_Reg_ therapy to gain acceptance in the clinic.

In this study we show that expanded human nT_Regs_ can be reconstituted in immunodeficient NOD-scid IL2rγc^null^ mice by providing a source of IL-2 to sustain their survival and expansion. We were able to achieve long-term human T_Reg_ reconstitution with just a single hydrodynamic injection of hu-IL2 expressing plasmid. Human T_Regs_ recovered from the animals retained the signature T_Reg_-specific phenotypic markers. Further, repopulation of T_Regs_ could be observed in multiple organs, including liver and lungs. More importantly, *in vivo* reconstituted T_Regs_ remained functionally intact and they could potently inhibit GVHD symptoms and prevent lethality in a new IL-2 accelerated xenogenic GVHD model.

IL-2/IL-2R pathway is known to play a critical role in peripheral maintenance and fitness of T_Regs_ [4]. Thus it is not surprising that in the absence of exogenous IL-2, we observed no T_Reg_ repopulation whatsoever, despite the lymphopenic milieu of NOD-scid IL2rγc^null^ mice, which should be conducive to homeostatic proliferation of lymphocytes. However, repopulation of human nT_Regs_ in multiple organs was achieved after a single hydrodynamic injection of human IL-2 expressing plasmid. This brings up the question of whether T_Reg_ therapy in human transplant settings would also require supplemental IL-2 as the cytokine is likely to be deficient in the lymphopenic environment created by lymphoablation therapy preceding HSC or other transplants. It is possible that unlike the murine milieu, other cytokines like human IL-15 and IL-7 that are secreted by non-T cells may be sufficient to drive the homeostatic proliferation of T_Regs_ to functionally effective numbers after infusions in humans. Our study also shows that although the IL-2 levels decline in the animals over time, the residual levels were sufficient for long-term reconstitution of T_Regs_ that retained efficient suppressive activity.

IL-2 is also a critical cytokine for the amplification of immune effector cells *in vivo* [19]. Thus it is not surprising that human PBMC transfer into NOD-scid IL2rγc^null^ mice in the presence of IL-2 resulted in a dramatic acceleration of GVHD with comparatively fewer numbers of cells. In addition, this accelerated disease development was achieved without the need for irradiation of the animals prior to the PBMC transfer. Massive infiltration of lymphocytes was observed in the spleen, lung and liver, reminiscent of the pathophysiology of allogenic GVHD in HLA-mismatched human transplants [20]. Thus, PBMC transfer with provision of a source of IL-2 *in vivo* led to the development of a highly sensitive and reproducible xenogenic GVHD model, which would be useful for evaluating immunosuppressive therapeutic regimens, including T_Regs_ therapy.

In this IL-2 stimulated accelerated GVHD model, it was clear that amelioration of the disease occurred only when the reconstituted T_Regs_ were adoptively co-transferred with the PBMCs. Although the data shows that the T_Regs_ proliferated *in vivo* in presence of IL-2 as assessed by CFSE dilution, it is possible that the level did not reach the critical threshold required for protection. The requirement of a threshold level of T_Regs_ for suppressing GVHD was evident in the adoptive transfer setting in which, T_Regs_ were able to suppress GVHD symptoms after co-transfer at a higher 1∶1 but not at the lower 1∶5 (T_Regs_: PBMCs) ratio. Transfer of equivalent numbers of T_Regs_ and effector cells have also been shown to be necessary for silencing GVHD in murine models [17]. This underscores the requirement for sufficient numbers of T_Regs_ to be infused for reliable therapeutic benefit in human transplant settings. Suboptimal numbers may be the reason for the fairly modest efficacy of T_Regs_ in ameliorating GVHD symptoms in a recent Phase I clinical study that examined the safety and efficacy of *ex vivo* expanded umbilical cord blood-derived T_Regs_ in 23 patients receiving allogenic cord blood HSC transplantation [21]. Low dose IL-2 supplementation could be considered as a strategy to enhance further T_Regs_ expansion *in vivo* but this would require a balancing act as the cytokine could also result in the amplification of effector cell proliferation leading to exacerbation of GVHD symptoms and graft rejection. On the positive side, the Phase I/II clinical studies in HSC transplant recipients have shown that T_Regs_ therapy is well tolerated with little or no infusion associated toxicity [21].

Another concern for using T_Regs_ for therapy is the perceived plasticity of the cells, which poses potential risk of their conversion to effector T cells that can themselves play a pathogenic role in GVHD induction or severity. Studies in mouse models have yielded mixed reports on the vulnerability of T_Regs_ to Th17 cell conversion under inflammatory conditions *in vivo*. Using Foxp3-reporter mouse strain, one group found that adoptively transferred T_Regs_ downregulate their Foxp3 expression and become capable of IL-17 secretion under inflammatory conditions [22] whereas another group reported that adoptively transferred T_Regs_ retained Foxp3 expression for several months under normal conditions [23]. In the presence of IL-2, human ovarian cancer-associated CD4^+^ regulatory T cells have also been shown to convert into proinflammatory IL17-producing helper T cells *in vitro* [13]. Hence we examined the possibility of *in vitro* expanded T_Regs_ converting to Th17 after *in vivo* transfer. We did not find detectable levels of IL-17 in the serum of animals reconstituted with nT_Regs_ in presence of IL-2. Although other cytokines that are upregulated under proinflammatory conditions may be required for reprogramming different subsets of T_Regs_ to Th17 cells *in vivo*, this appears unlikely as both cultured and reconstituted population of T_Regs_ could suppress GVHD lethality upon co-transfer with PBMCs at the optimal 1∶1 ratio. This is also borne out by studies showing that nT_Regs_ isolated and expanded *in vitro* do not convert to Th17 phenotype [5]. Moreover, we did not detect IL-17A even in nT_Reg_ reconstituted animals that developed GVHD.

The data we presented here provide evidence for the *in vivo* engraftment capability of expanded human nT_Regs_. These findings hold important implications for studying the *in viv*o function of human nT_Regs_ and allow researchers to develop new methods to improve the *in vivo* reconstitution of human nT_Regs_ without affecting their suppressive function. Finally, we believe that, this novel hu-T_Reg_ mouse model offers a pre-clinical model to study the *in vivo* function and stability of human nT_Regs_ and their ability to modulate autoimmune diseases and GVHD.

## Materials and Methods

### Isolation and Expansion of nT_Regs_

Human nT_Regs_ were purified from buffy coat in two steps as described previously [5]. In the first step CD4^+^ T cells were enriched by negative selection using a cocktail of nine monoclonal antibodies. In the second step CD25^+^ cells were isolated by positive selection from purified CD4^+^ cells using anti-CD25 antibody in a Robosep instrument (Stem cell Technologies, Vancouver, BC, Canada). Isolated nT_Regs_ were expanded in the presence of CD3/CD28 T-cell expander dynabeads (3∶1 ratio), rhIL-2 (1000 U ml^−1^) and rapamycin (100 ng ml^−1^) for 19 ± 1 days and at the end of the culture period, the beads were removed and the cells were used for testing phenotype, suppressive function and reconstitution.

### Animals

NOD-SCID IL2rγc^null^ mice were obtained from Jackson Laboratory (Bar Harbor, ME) and maintained in specific pathogen free conditions at the Paul L. Foster School of Medicine, TTUHSC animal facility. All the experiments were done with 5–6 week old mice using study protocols approved by the TTUHSC IRB and IACUC committees.

### Hydrodynamic Injection of Plasmids Expressing hIL-2

Human IL-2 gene was PCR amplified from PHA stimulated human PBMCs and cloned into the Adeno associated virus vector (pAAV-IRES-hrGFP) obtained from Agilent Technologies (Santa Clara, CA, USA). Plasmid DNA was purified using the endotoxin free maxi prep-kit from Qiagen (Valencia,CA,USA). For expressing hIL-2 *in vivo*, NOD-scid IL2rγc^null^ mice were injected with 70 µg of plasmid using 27-gauge needle in a volume of saline equivalent to 8% of the body mass of the mouse [24]. The total volume was delivered within 5–8 seconds and animals were bled periodically through retro-orbital puncture to measure serum cytokine levels.

### T_Regs_ Reconstitution and Isolation of T_Regs_ from Reconstituted Animals

5–6 week old IL-2 expressing and control NOD-scid IL2rγc^null^ were iv injected with 5–10×10^6^ *in vitro* expanded nT_Regs_ and animals euthanized on different days and single cell suspensions prepared from blood, spleen, liver and lungs by standard procedures. Human cells were enriched from T_Regs_ reconstituted animals by negative selection with mouse/human chimera enrichment Kit (Stem Cell Technologies) for use in suppression assays and for adoptive transfer experiments.

### Antibodies and Flow Cytometry

Various fluorescent conjugated antibodies to human CD4, CD25, CD27, CD45, CD127, CTLA4, PD1 and Foxp3 were from BD Biosciences. Cells were surface stained first using appropriate combinations of mAbs, and either used directly for flow cytometric analyses or further processed for intracellular staining with CTLA4 and Foxp3 antibody. All stained samples were subsequently analyzed on FACS Canto II instrument (BD) using FlowJo software.

### T_Regs_ Suppression Assay

1×10^6^ CD4^+^CD25^−^ (responder) cells were labeled with carboxyfluorescein succinimidyl ester (CFSE, Invitrogen, Carlsbad, CA, USA) at a concentration of 1 µM/1×10^6^ cells for 10 min at 37°C. Labeling was terminated by adding 5 volumes of ice cold RPMI medium containing 10% FBS and incubating on ice for 5 minutes. After 3 washes in complete RPMI medium, cells were cultured alone or with unlabeled *in vivo* or *in vitro* expanded nT_Regs_ at various (responder : T_Regs_) ratio in the presence of anti-CD3/CD28 coated micro beads at a ratio of 50∶1 (responder cells: beads). CD4^+^CD25^−^ cells in medium alone served as control. After 5 days of culture at 37°C under 5% CO_2_, the micro beads were removed and cells washed twice before analyzing cell division in various culture conditions. Cells undergoing division were identified by the decrease in CFSE, resulting from dilution of dye with each division.

### ELISA for Serum IL-2 and IL-17A

To determine the IL-2 and IL-17A levels in the serum, animals were bled periodically through retro-orbital puncture. IL-2 or IL-17A serum levels were determined using commercial ELISA kits according to manufactures instructions (Biolegend Inc, San Diego, CA, USA).

### Induction of Xenogenic GVHD

Human PBMCs were injected through tail vein (0.5–5×10^6^/mouse) into control and hIL-2 expressing animals. In some experiments, PBMCs were mixed with pooled T_Regs_ isolated from spleens of animals reconstituted for 12 days. The animals were monitored daily for symptoms of GVHD, including hunched back, ruffling of hair, diarrhea and weight loss. Animals were sacrificed when they lost 20% body weight.

## Supporting Information

Figure S1 Phenotypic and functional characterization of *in vitro* expanded human nT_Regs._ (A) CD4^+^CD25^+^ cells were expanded *in vitro* as per protocol and were harvested on day 19 and surface stained for CD4, CD25, CD27, CD127 expression and intracellular staining was carried out for CTLA4 and Foxp3 expression. Data shown are one representative of three independent experiments and numbers indicate the percentage of positive cells. (B) Functional stability of T_Regs_ expanded *in vitro* as determined by CFSE dilution of CD4^+^CD25^−^ responder cells cultured with medium alone (a), stimulated in the absence of T_Regs_ (b) and stimulated in the presence of *in vitro* expanded T_Regs_ (c). One representative result from three independent experiments is shown.
(TIF)
