# Supplementary figures and images for: Long-Term Engraftment of Human Natural T Regulatory Cells in NOD/SCID IL2rγcnull Mice by Expression of Human IL-2
Source: PLoS One. 2012 Dec 18;7(12):e51832. doi: 10.1371/journal.pone.0051832 (PMC3525660; doi:10.1371/journal.pone.0051832)

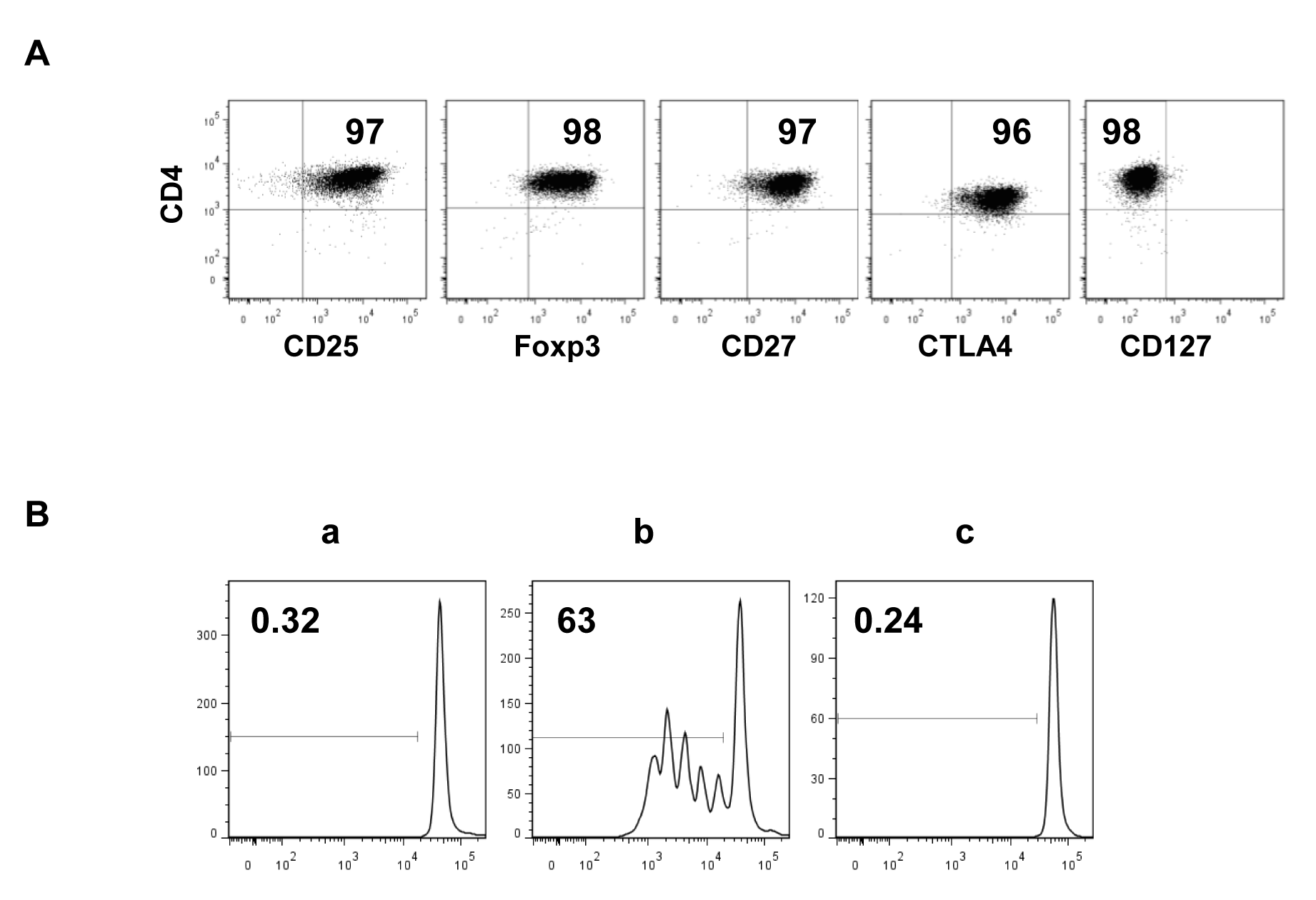

Supplement: Figure S1 — Phenotypic and functional characterization of in vitro expanded human nTRegs. (A) CD4+CD25+ cells were expanded in vitro as per protocol and were harvested on day 19 and surface stained for CD4, CD25, CD27, CD127 expression and intracellular staining was carried out for CTLA4 and Foxp3 expression. Data shown are one representative of three independent experiments and numbers indicate the percentage of positive cells. (B) Functional stability of TRegs expanded in vitro as determined by CFSE dilution of CD4+CD25− responder cells cultured with medium alone (a), stimulated in the absence of TRegs (b) and stimulated in the presence of in vitro expanded TRegs (c). One representative result from three independent experiments is shown. (TIF) [file pone.0051832.s001.tif]
